# Prevalence of tuberculosis drug resistance in 10 provinces of China

**DOI:** 10.1186/1471-2334-8-166

**Published:** 2008-12-11

**Authors:** Guang Xue He, Yan Lin Zhao, Guang Lu Jiang, Yu Hong Liu, Hui Xia, Sheng Fen Wang, Li Xia Wang, Martien W Borgdorff, Marieke J van der Werf, Susan van den Hof

**Affiliations:** 1 National Center for TB control and prevention, China Center for Disease Control and Prevention (CDC), Beijing, PR China; 2 National Tuberculosis Reference Laboratory of China CDC, Beijing, PR China; 3 KNCV Tuberculosis Foundation, The Hague, the Netherlands; 4 Academic Medical Center, University of Amsterdam, Amsterdam, the Netherlands

## Abstract

**Background:**

The emergence of drug-resistant tuberculosis (TB) hampers TB control. Ten provinces in China performed drug resistance surveys among tuberculosis (TB) patients in 1996–2004 to assess levels of drug resistance.

**Methods:**

Provincial drug resistance surveys included all isolates from newly diagnosed, smear-positive TB patients. Drug susceptibility testing (DST) against isoniazid, rifampicin, streptomycin and ethambutol was carried out in the provincial laboratories. For purposes of quality assurance, a random sample (11.6%) was re-tested by the national reference laboratory (NRL).

**Results:**

Of 14,059 patients tested 11,052 (79%) were new TB cases. The weighted mean prevalence of multi-drug resistant tuberculosis (MDR-TB) among all cases was 9.3% (range 2.2%–10.4%); 5.4% (range 2.1% – 10.4%) among new cases and 25.6% (range 11.7%–36.9%) among previously treated cases. Adjusting the drug resistance proportions using the re-testing results did not change the estimated national mean prevalence significantly. However, in some individual provinces the estimated resistance proportions were greatly influenced, especially among re-treatment patients.

**Conclusion:**

MDR-TB levels varied greatly between provinces in China, but on average were high compared to the global estimated average of 4.8%. This study shows the importance of quality-assured laboratory performance. Programmatic management of drug-resistant TB, including high quality DST for patients at high risk of resistance and treatment with second-line drugs, should become the standard, especially in high MDR-TB settings.

## Introduction

The emergence of resistance to drugs used to treat tuberculosis, and particularly multi-drug resistant tuberculosis (MDR-TB) [1], has become a significant public health problem in a number of countries and an obstacle to effective global TB control. The emergence of drug resistant *Myobacterium tuberculosis *is associated with ineffective treatment of tuberculosis, leading to acquired resistance and transmission of drug-resistant strains. With an estimated MDR-TB proportion of 4.8% among incident TB cases globally, almost half a million (489,139 (95% CI 455,093–614,215)) cases of MDR-TB are estimated to emerge world-wide every year [1].

Treatment of MDR-TB, requires use of costly, toxic and less effective second-line drugs for at least 18 months because the bacilli are resistant to the most effective first-line drugs rifampcin and isoniazid [2]. In case of extensively drug-resistant TB (XDR-TB), i.e. MDR-TB with additional resistance to any fluoroquinolone and at least one of the three injectable second-line anti-TB drugs (i.e. amikacin, kanamycin, capreomycin), treatment options are seriously limited[3]. The first estimates indicate that about 10% of all MDR-TB isolates meet the criteria for XDR-TB [4].

China has the second largest number of TB cases in the world [5], and also is one of the countries with high levels of drug resistant TB [1]. Based on the data of the 4th national TB epidemiological survey in 2000 [6], it is estimated that there were 4.5 million prevalent TB cases in China, of which 1.96 million were pulmonary, bacteriologically confirmed cases. The observed MDR-TB prevalence was 10.7%, so it was estimated that there were 209,720 (95% CI 149,159–270,841) cases of pulmonary, bacteriologically confirmed MDR-TB. The magnitude and pattern of drug resistance may vary per region because of the huge size of the country, the diverse population density, and the unbalanced economic development in China. However, the sample size of the national survey was too small to be able to stratify the data per province.

In order to obtain insight into the prevalence and distribution of resistance to anti-TB drugs, China has joined the global project on anti-tuberculosis drug resistance surveillance organized by World Health Organization and International Union Against Tuberculosis and Lung Disease (WHO/IUATLD) [1]. By 2007, 13 out of 31 provinces with sufficient laboratory quality and capacity have conducted drug resistance surveys. Ten provinces have obtained final results, covering 38% (483 million out of 1.27 billion inhabitants) of the total Chinese population. Results have been published before, but not in the international scientific literature [1,7-15]. Here we give an overview of the results of the drug resistance surveys conducted in ten provinces in China between 1996 and 2004. Moreover, we have adjusted the estimates taking into account re-testing results of a random sample (11.6%) of all isolates from those provincial surveys.

## Methods

### Sampling method

For all surveys, the number of new smear positive cases to be included per province was calculated according to the sampling method in "*Guidelines for surveillance of drug resistance in tuberculosis" *developed by WHO/IUATLD [16], whereby the precision was set at 1.5%, and the initial drug-resistance rate of one drug in past survey was set at 2.7% based on the proportion of rifampicin resistant isolates among new patients in the 1990 national TB epidemiological survey [6]. As recommended, this sample size was multiplied by 2 to take into account the cluster sampling method, and 15% was added to take losses into account. The required intake period was estimated based on the number of newly diagnosed new smear positive cases in the previous year. All previously treated cases diagnosed during the inclusion period were also included in the surveys.

Beijing and Shanghai municipalities included all smear-positive cases occurring, while the other provinces used cluster-based sampling. In these province 30 (40 in Guangdong) counties or districts were randomly selected, and all smear-positive cases diagnosed in these sites during the study period were included. Table 1 shows the basic sampling information of the 10 provinces that have finished a survey. Henan province has performed two surveys, data from both rounds are included in this overview.

**Table 1:** Sampling information for the 10 provinces of which final results of one or more drug resistance surveys are available in 2008

Province	Year	Population (*million)	No. of counties/districts	No. of reported SS+ cases in year of survey	No. of survey sites	New cases		Previously treated cases	
						n	(%)	n	(%)
Henan	1996	91.0	157	11,822	30	646	(47)	726	(53)
Shandong	1997	86.5	141	18,488	30	1009	(82)	220	(18)
Zhejiang	1998	43.9	89	6,278	30	809	(85)	145	(15)
Guangdong	1998	69.0	113	26,200	40	1482	(90)	166	(10)
Hubei	1999	57.9	88	23,633	30	859	(78)	238	(22)
Liaoning	1999	40.9	100	8,002	30	818	(90)	86	(10)
Henan	2001	95.6	157	14,680	30	1222	(82)	265	(18)
Inner Mongolia	2001	23.8	101	3,564	30	876	(69)	386	(31)
Beijing	2004	14.2	18	730	All	1043	(87)	154	(13)
Shanghai	2004	13.3	19	2,958	All	764	(79)	200	(21)
Heilongjiang	2004	38.1	87	21,560	30	1574	(79)	421	(21)

The results described here are marginally different from those reported in the WHO/IUATLD report on drug resistance [1], due to the fact that for some provinces incomplete results were reported to WHO.

### Intake of patients

In all surveys, each newly registered TB patient, positive on sputum smear microscopy, was interviewed by the clinician using the standard WHO questionnaire to obtain the treatment history. The treatment history of included cases was classified by medical staff into new and previously treated cases using the WHO questionnaire for interviewing patients and by checking the available medical documents of the patient. In all surveys, new patients were defined as patients who had never received anti-tuberculosis treatment or had received previous treatment for less than a month. Previously treated patients were defined as those having previously received anti-TB treatment for more than 30 days. Patients in Henan (1996) and Liaoning (1999) were reinterviewed to check the treatment history after DST results became available and showed unexpectedly high MDR-rates among new cases.

### Laboratory methods

The two smears with the highest bacterial counts were cultured, and one culture was submitted for DST. Smear microscopy and culture was performed in the county or district laboratory (except for the 1996 Henan survey, where culture was done at the provincial level), while drug resistance testing was done at the provincial level. Ziehl-Neelsen staining was used for sputum smear microscopy. Sputum smear microscopy positive samples were cultured on Lowenstein-Jensen (LJ) culture medium. *M. tuberculosis *complex was identified by also culturing on LJ medium containing p-nitrobenzoic acid (PNB), where growth indicates the bacilli which are not part of the *M. tuberculosis *complex. The samples containing non-tuberculosis mycobacteria were excluded from this study. Drug susceptibility testing was performed on LJ medium impregnated with isoniazid (INH), rifampicin (RIF), streptomycin (SM), and ethambutol (EMB) according to the proportion method as recommended by WHO/IUATLD [1,16]. The concentration of anti-TB drugs used was 0.2 mg/L for INH, 40 mg/L for RIF, 2 mg/L for EMB, and 4 mg/L for SM. Multi-drug resistant (MDR) TB was defined as isolates being resistant to both RIF and INH.

### Laboratory quality control

External QA for smear and culture in county and district level laboratories was conducted by the prefectural and provincial TB laboratories based on the WHO guideline [16]. For internal quality assurance (QA) on DST, a standard H37Rv strain was included for each new batch of LJ medium. External QA on DST included proficiency testing of the National Reference Laboratory (NRL) and provincial reference laboratories before the start of the survey by the supranational laboratory (SRL), and blinded re-testing of a random selection of approximately 10% of the isolates from each province by the NRL of China during the survey. Before 2002, the serving SRL for China was the Korean Institute of Tuberculosis; afterwards the Public Health Laboratory Hong Kong fulfilled this function.

### Data management and analysis

Data were double entered and discrepancies were checked against the raw data. The proportion of resistant isolates per province was calculated. The weighted mean prevalence of resistance for the ten provinces was calculated by weighting the observed prevalence of resistance by the number of notified smear-positive TB cases per province in 2006. For Henan province, only the estimates from the most recent survey (2001) were used.

For the isolates retested by the NRL, aggregated results on concordance between provincial and NRL DST results were available. We calculated concordance for all isolates together and separately for the isolates considered susceptible and for the isolates considered resistant to a particular drug according to the provincial laboratory. Comparative results of the NRL after re-testing about 10% of the isolates per province were used to estimate the adjusted provincial resistance proportions per drug, for new, previously treated and combined cases separately. To this end, the ratio of the proportion of resistant strains among all re-tested strains according to the provincial laboratory over the proportion of resistant strains among all re-tested strains according to the NRL was calculated. A ratio over unity indicates that the provincial laboratory overestimated the resistance proportion, and a ratio below unity indicates it underestimated resistance. The proportion of resistant strains among all isolates tested by the provincial laboratory was divided by this ratio to estimate the adjusted provincial resistance proportions to the individual drugs. The ratio was applied separately to new and previously treated cases. This estimation assumes that the results of the NRL are the gold standard, that the 10% sample re-tested is representative of all isolates with regard to observed differences between the provincial and reference laboratory.

### Ethical issues

All provincial surveys were approved by the Ministry of Health of China. Patients were asked for written informed consent before interviewing, and collecting three sputum samples. The DST results were communicated to the treating physicians. The decision to change the treatment based on the DST results was left to the treating physician.

## Results

The total number of patients included in the eleven drug resistance surveys in ten provinces was 14,059, of which 11,052 (79%) were new TB cases (Table 1).

### Drug resistance

Drug resistance levels are shown in Table 2. The weighted mean prevalence of any drug resistance in the ten provinces was 24.3% (range 14.8% – 42.1%) among new cases and 51.8% (range 27.5% – 67.5%) among previously treated cases.

**Table 2:** Observed drug resistance in new, previously treated (PT) and all tuberculosis cases in different provinces in China

Province	Resistance to																	
	Any drug			Rifampcin			Isoniazid			Ethambutol			Streptomycin			MDR-TB		
	New	PT	All	New	PT	All	New	PT	All	New	PT	All	New	PT	All	New	PT	All
Henan (1996)	35.0	66.0	51.4	14.6	43.5	23.9	24.0	48.8	37.1	7.7	18.9	13.6	26.0	50.7	39.1	10.8	34.4	23.3
Shandong	17.6	50.0	23.4	3.8	23.2	7.2	11.3	40.5	22.4	1.7	10.5	6.8	12.2	34.5	25.9	2.9	19.6	5.9
Zhejiang	14.8	59.3	21.6	6.4	44.4	12.2	11.2	51.4	17.5	4.7	18.0	6.8	8.9	34.2	12.8	4.5	34.5	9.0
Guangdong	18.0	33.7	20.2	7.1	19.9	8.7	11.4	22.3	12.5	3.6	7.8	4	9.3	16.9	10.1	5.3	15.7	6.6
Hubei	17.5	44.5	23.3	3.8	26.9	8.8	9.7	33.2	14.8	0.6	8.8	2.4	11.4	25.6	14.5	2.1	21.8	6.4
Liaoning	42.1	55.8	43.3	11.4	29.1	13.1	25.3	41.9	26.9	3.8	14.0	4.8	34.1	41.9	34.8	10.4	24.2	11.7
Henan (2001)	29.8	60.8	35.5	9.6	42.6	15.5	17.0	47.2	22.4	4.3	18.1	6.8	22.2	43.0	25.9	7.8	36.6	12.9
Inner Mongolia	35.7	65.3	44.8	9.6	45.3	20.5	20.3	56.5	30.3	8.9	31.8	15.3	21.3	29.9	23.7	7.0	36.8	16.1
Beijing	17.9	35.1	20.1	4.2	14.9	5.6	8.7	24.7	10.8	4.1	9.1	4.8	9.1	21.4	10.7	2.3	11.7	3.5
Shanghai	15.4	27.5	17.9	4.8	15.0	7.0	11.7	21.5	13.3	3.0	10.0	4.5	8.1	12.5	9.0	3.9	12.5	5.7
Heilongjiang	36.1	67.9	42.8	10.6	40.4	16.9	16.9	48.0	23.6	5.9	24.5	9.8	24.3	32.3	26.0	7.2	30.4	12.1
Weighted mean*	24.3	51.8	29.5	7.2	31.5	11.8	14.0	39.2	19.6	3.3	14.5	6.0	16.4	31.1	20.5	5.4	25.6	9.3

* the weighted mean for the 10 provinces excludes 1996 Henan data

There were considerable differences in resistance proportions between provinces for the individual drugs, but the trend was the same in all provinces. In new cases, on average resistance proportions were highest for streptomycin (16.4%) and isoniazid (14.0%), intermediate for rifampicin (7.2%) and lowest for ethambutol (3.3%). In previously treated cases, average resistance proportions were lowest for ethambutol (14.5%), intermediate for streptomycin (31.1%) and rifampicin (31.5%), and highest for isoniazid (39.2%).

**Figure 1:**
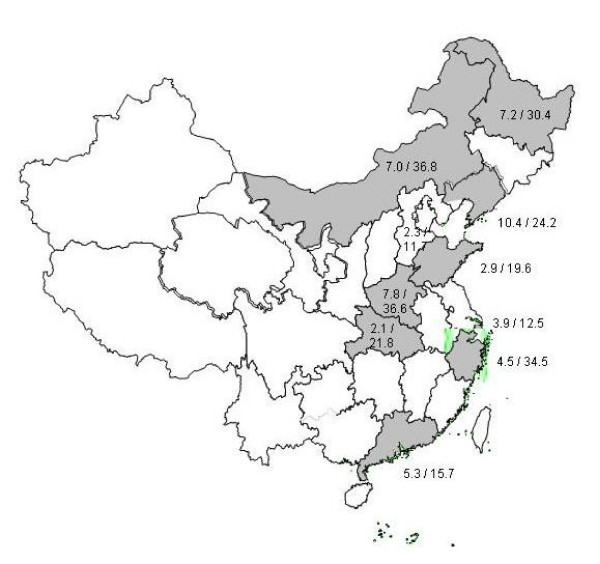
MDR-TB proportions among new and previously treated cases observed in the provincial drug resistance surveys.

The weighted mean MDR prevalence was 5.4% (range 2.1% – 10.4%) among new patients and 25.6% (range 11.7% – 36.8%), among previously treated patients. The individual MDR prevalence among new and previously treated cases are shown in Figure 1. Four out of the ten provinces observed over 6.5% of MDR among new cases, with this cut-off previously being defined as a MDR hotspot [2]. The four provinces Henan (in both surveys), Zhejiang, Inner Mongolia and Heilongjiang reported MDR prevalences of over 20% in previously treated cases.

Both in Henan (1996 survey) and Liaoning (1999), reinterviews of patients indicated that an estimated 25–30% misclassification of previously treated cases as new cases had occurred, so the prevalence of MDR among new TB cases is more likely to be around 11% instead of 16% in Henan (1996) [1,17] and 8% instead of the reported 10% in Liaoning [1].

### Re-testing results

**Figure 2:**
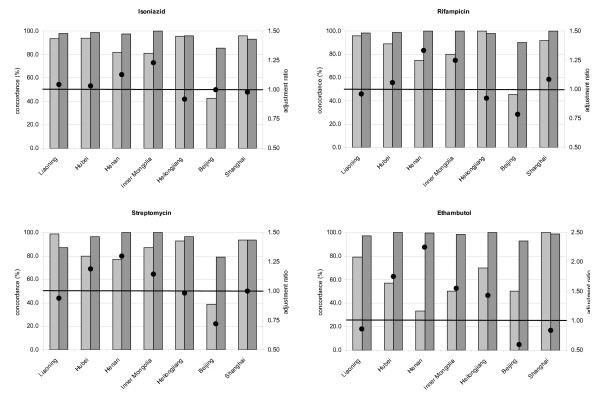
**Re-testing results by the national reference laboratory on a random selection of the isolates as tested by the provincial laboratories.** Concordance between provincial laboratories and the national reference laboratory for isolates tested resistant by the provincial laboratory is shown by light bars, for isolates tested susceptible by dark bars. The adjustment ratio (•) indicates the amount of over- or underestimation of resistance by the provincial laboratory (ratio above unity indicates overestimation).

The re-testing results on drug resistance by the NRL were available for the seven surveys performed in and after 1999. A total number of 1,033 isolates were re-tested, reflecting 11.6% of the isolates from these seven surveys. The number of provincial re-tested isolates varied between 94 and 268. Overall concordance between the provincial and national DST results on whether an isolate was resistant or susceptible was quite high: the median was 94.7%, 97.1%, 93.8% and 97.1%, for INH, RIF, SM and EMB, respectively. Concordance for susceptibility was usually over 90% in the provincial laboratories (Figure 2): the medians were 97.7%, 98.9%, 96.3%, and 98.9%, respectively. However, concordance for resistance was rather low for several drugs in several provincial laboratories: the medians were 93.4%,, 88.9%, 87.2% and 57.1%, respectively. As a consequence, Hubei, Henan and Inner Mongolia provincial laboratories overestimated resistance proportions for all four drugs while Liaoning, Heilongjiang and Beijing provinces underestimated resistance proportions for three out of four drugs. In Shanghai laboratories, RIF resistance was overestimated and EMB resistance underestimated (see Figure 2).

The weighted means of prevalence of drug resistance proportions over all provinces, based on re-testing by the NRL, were hardly affected, but in some provinces resistance proportions were greatly influenced. In Henan (2001) and Inner Mongolia, adjusted resistance proportions for RIF, the most important predictor for MDR, decreased by approximately 2% in new cases and 10% in previously treated cases. For previously treated cases, the estimated RIF resistance proportions decreased from 43% and 45% to 32% and 36% respectively. On the other hand, RIF resistance proportions were underestimated in Beijing laboratories: taking into account re-testing results, RIF resistance was adjusted from 4.2% to 5.3% in new cases and from 14.9% to 19.0% in previously treated cases. Due to relatively small numbers, these differences will not be statistically significant, but they do indicate the direction and magnitude in which resistance proportions were under- or overestimated in the different provinces.

## Discussion

The results of the provincial drug resistance surveys show that the prevalence of drug resistance varied greatly between the provinces, but on average was worryingly high with a weighted mean for MDR-TB of 9.3% among all cases; 5.4% among new cases and 25.6% among previously treated cases. The global MDR-TB estimates are 4.8% for all cases, 3.1% for new cases and 19.3% for previously treated cases. Provinces with the highest resistance rates were Henan, Liaoning, Inner Mongolia and Heilongjiang. The latter two are bordering the former Russian Federation, where resistance levels are known to be extremely high [1,18]. and Liaoning is bordering these two provinces. Henan however, is located in the middle of China and borders two provinces with clearly lower MDR-TB levels. Therefore, it is necessary to gain insight into the distribution of drug resistance levels in all provinces of China.

What is new in the results presented in this paper is that we adjusted estimates of drug resistance based on re-testing by the NRL. Unfortunately, retesting results on an individual level were not available. With the available aggregated results we were able to show that overall high agreement between resistance proportions may conceal insufficient agreement for resistant and susceptible isolates separately. As provincial results counterbalanced each other, the adjusted estimates hardly affected the estimated national mean prevalence of drug resistance. However, in some individual provinces resistance proportions were greatly influenced. Adjustments were proportional, therefore, resistance percentages in previously treated cases were affected the most in absolute figures as in this group resistance percentages were highest. As resistance patterns for individual isolates in the provincial and national reference laboratory were not available, it was not possible to estimate adjusted MDR prevalences. However, as rifampicin resistance has been proven to be a good marker for MDR in many countries, provincial MDR levels will need to be adjusted in the same direction.

In Henan (1996) and Zhejiang, the then running policy was that re-treatment was not fully free of charge. This may have precluded patients from disclosing their treatment history. As a consequence, results from the two surveys in Henan may not be directly comparable. Surveys following the Liaoning survey in 1999 have had more elaborate checking of treatment status built in and in general survey protocols have been more closely followed with regard to both survey methods and laboratory methods. In the period 1992–2001, China has implemented the TB Control Project, funded by a World Bank loan. This project covers 13 provinces, and provides treatment free of charge to both new and previously treated cases. Since 2002, drugs are also free of charge for new cases and cases with one previous treatment episode in the other provinces. For smear-negative cases treatment is free of charge in China from 2005 onwards.

Drug resistance proportions among new, and previously treated TB cases are important indicators for TB epidemiology. In a well functioning TB control program with low levels of defaulting from treatment, high resistance levels are expected among previously treated cases because drug resistance is a strong risk factor for recurrent TB. This is what we observed in China. However, if a good TB control program is in place, also the proportion of previously treated patients among all TB patients should be low. In China the proportion of previously treated patients was high on average, about 20%. Globally, the proportion of previously treated patients is currently estimated to be 11% [5]. There is a well established relationship between the proportion of retreatment cases and proportion of MDR among new cases [8]. In our data we observed a weak positive linear association (R^2 ^= 0.18).

Many possible explanations for the development of drug resistance in China exist, and different explanations may prevail in different areas of this vast country. These include the inadequate use of anti-TB drugs in public hospitals, lack of supervision of treatment, poor drug management, and absence of infection control measures in hospitals. Availability of anti-TB drugs without a prescription in China in the past may have contributed to the development of drug resistance.

In a situation where drug resistance testing is not done routinely, as currently is the case in China, drug resistance surveys are good tools to determine the magnitude of the problem, meanwhile building laboratory capacity and establishing continuous drug resistance surveillance. The results should be the basis for development and implementation of interventions to reduce the problem of resistance. In China, the results have contributed to introduction of programmatic management of drug-resistant TB (PMDT), using the GLC mechanism (Green Light Committee for Access to second-line anti-tuberculosis drugs), and supported by the Global Fund (GFATM). It has been piloted in two provinces and a rapid expansion in 14 more provinces is under preparation with a total of 15,011 MDR-TB cases in these 16 provinces.

In conjunction with the drug resistance surveys, a quality assurance system including yearly proficiency testing of provincial laboratories is evolving in China [16,17]. Therefore, we expect to find better agreement on DST results between provincial laboratories and the NRL in future drug resistance surveys within China. Improved laboratory quality for DST obviously is also important for individual patient management.

China is currently conducting a nationwide anti-TB drug resistance survey. This will give insight into the overall level of drug resistance in China. However, as the sample size per province is too small for precise estimates of resistance per province, it is also necessary to perform separate drug resistance surveys in the provinces that have not done so yet. As there is a wide variation in observed drug resistance between provinces, it is important that all provinces in China conduct their own surveys, and repeat surveys to allow for evaluation of trends. Provincial surveys should also allow for differentiation between the different retreatment categories (relapse, retreatment after default, retreatment after failure). In future surveys, the rate of XDR-TB in MDR-TB cases should also be determined in order to inform MDR-TB treatment design.

## Conclusion

Resistance levels vary greatly within China and are unknown for a large part of the TB patient population. However, on average MDR-TB levels are worryingly high. PMDT, including routine, quality-assured DST for those patients at high risk of resistance, especially failure cases, and programmatic treatment with second-line drugs, should become integrated on a routine basis within TB control in the whole country of China, with priority for high MDR-TB settings.

## Competing interests

The authors declare that they have no competing interests.

## Authors' contributions

GXH participated in the design and coordination of the study, was responsible for data collection, analysis and interpretation of the data and drafted the manuscript. YLZ participated in design and coordination of the study, performed quality control at provincial laboratories, and helped to draft the manuscript. GLJ collected data, and was involved in re-testing. YHL was involved in quality control and re-testing. HX was involved in re-testing and reviewed the manuscript. SFW was involved in quality control and retesting. LXW participated in the design and coordination of the study and reviewed the manuscript. MWB assisted in interpretation of the data and critically reviewed the manuscript. MJW assisted in interpretation of the data and critically reviewed the manuscript. SH assisted in analysis and interpretation of the data, and assisted in drafting the manuscript.

## Pre-publication history

The pre-publication history for this paper can be accessed here:


